# Size-Dependent Alloying Ability of Immiscible W-Cu Bimetallic Nanoparticles: A Theoretical and Experimental Study

**DOI:** 10.3390/nano11041047

**Published:** 2021-04-20

**Authors:** Hongbo Zhang, Tao Liu, Siqi Zhao, Zhanyuan Xu, Yaozha Lv, Jinglian Fan, Yong Han

**Affiliations:** State Key Laboratory for Powder Metallurgy, Central South University, Changsha 410083, China; zhanghb1201@163.com (H.Z.); 203020@csu.edu.cn (T.L.); zhaosiqi@csu.edu.cn (S.Z.); 153301006@csu.edu.cn (Z.X.); lvyaozha@163.com (Y.L.)

**Keywords:** nanoscale size effect, alloying ability, immiscible W–Cu systems, bimetallic nanoparticles, bond energy model

## Abstract

The preparation of alloyed bimetallic nanoparticles (BNPs) between immiscible elements is always a huge challenge due to the lack of thermodynamic driving forces. W–Cu is a typical immiscible binary system, and it is difficult to alloy them under conventional circumstances. Here, we used the bond energy model (BEM) to calculate the effect of size on the alloying ability of W–Cu systems. The prediction results show that reducing the synthesis size (the original size of W and Cu) to less than 10 nm can obtain alloyed W–Cu BNPs. Moreover, we prepared alloyed W50Cu50 BNPs with a face-centered-cubic (FCC) crystalline structure via the nano in situ composite method. Energy-dispersive X-ray spectroscopy (EDS) coupled with scan transmission electron microscopy (STEM) confirmed that W and Cu are well mixed in a single-phase particle, instead of a phase segregation into a core-shell or other heterostructures. The present results suggest that the nanoscale size effect can overcome the immiscibility in immiscible binary systems. In the meantime, this work provided a high-yield and universal method for preparing alloyed BNPs between immiscible elements.

## 1. Introduction

For decades, bimetallic nanoparticles (BNPs) have attracted ever-increasing interest because of their important scientific significance and application prospect [1,2]. Unlike the monometallic systems, BNPs cause changes in structure and special physicochemical properties due to the alloying effect. For example, BNPs can form four possible mixing patterns: core-shell, subcluster segregated, mixed, and three shell [3]. Recently, BNPs have been used as high-efficiency catalysts to solve the energy problems faced by humankind [4,5]. However, many bimetallic systems are immiscible in the solid or liquid state; that is, the constituent elements cannot be spontaneously alloyed [6,7]. According to Miedema’s model, the formation enthalpy (also known as the enthalpy of mixing) is the main parameter to characterize the alloying ability of bimetallic systems [8]. That is, if the enthalpy of formation is negative, it can form alloys spontaneously. Moreover, the lower this value is, the stronger the alloying ability will be. Given this matter, researchers have found that particle size plays an important role in enhancing the alloying ability of immiscible BNPs. Numerous researchers have conducted in-depth researches on the size-dependent alloying ability in theories and experiments. For instance, Qi et al. proposed a bond energy model (BEM) to evaluate the size and sharp effect on the thermodynamic properties of BNPs [9,10]. They found that reducing the synthesis size (the original size of W and Cu) can alloy the immiscible bimetallic systems in the state of nanoparticles, e.g., Cu–Ag, Au–Ni, Ag–Pt, and Au–Pt. However, they did not experimentally verify the correctness of the theoretical predictions, nor did they explore bimetallic systems (although they are common) with a heterogeneous crystalline structure. Yang et al. synthesized a variety of immiscible Cu-based alloyed BNPs with a size below 25 nm by the high-temperature shock method and found that alloyed Cu-based nanoparticles have excellent catalytic performance. Nevertheless, their researches did not involve bimetallic systems with extremely large positive mixing enthalpies, and the yield of nanoparticles was also greatly limited by the preparation method.

W–Cu system is a familiar immiscible system with the highest mixing enthalpy (+35.5 KJ/mol) of almost all bimetallic systems. At the same time, the crystalline structure, melting point, and electronegativity of W and Cu are quite different. Bulk W–Cu materials are widely used in aerospace, military, electronic information, and other fields [11,12,13,14]. Conventional powder metallurgy methods do not solve the problem of the immiscibility of W and Cu, which results in the outcome that the overall properties of bulk W–Cu materials, such as poor ductility, are insufficient to meet industrial needs. As we all know, the properties of the final sintered bulk materials are determined by the performance of the powder particles. Therefore, there is an urgent demand to find a high-yield method for preparing alloyed W–Cu nanoparticles to improve the overall properties of the bulk W–Cu material. As mentioned above, it may be an effective way to solve the immiscibility of W and Cu by reducing the synthesis size. Therefore, there must be a critical size that can make alloying W–Cu BNPs for a given composition possible. It is worth noting that alloying refers to forming a single-phase solid solution due to the mutual dissolution of atoms, rather than just forming a core-shell structure or surface alloying. Unfortunately, there are few reports about the successful preparation of alloyed W–Cu BNPs, and there are almost no reports on the theoretical prediction of the influence of the synthesis size on the alloying ability of W–Cu BNPs.

Here, we use the BEM to study the size effect on the alloying ability of the W–Cu system and deduce the critical size required for alloying of a given composition. At the same time, with the help of theoretical derivation combined with the nano in situ composite method proposed in the early stage of our group [15,16,17], we successfully prepared single-phase W50Cu50 (at.%) BNPs.

## 2. Materials and Methods

### 2.1. Theoretical Calculation Procedures

The BEM is proposed by Qi, who comprehensively considers the influence of three factors of size, shape, and relaxation on the alloying ability of BNPs [9,18]. The details of the BEM are as follows:

According to the classical thermodynamic theory, the formation enthalpy can be used to predict the alloying ability of binary alloy systems [19]. For W–Cu bulk alloys, the formation enthalpy ($H_{b}^{WCu}$) equals

(1) $$H_{b}^{WCu}{=E}_{b}^{WCu}-\left(1-x\right)E_{b}^{W}{-xE}_{b}^{Cu}$$

where $E_{b}^{WCu}$ is the cohesive energy (CE) of the bulk W–Cu alloys, $E_{b}^{W}$ and $E_{b}^{Cu}$ refer to the CE of W and Cu, respectively, and *x* is the atomic concentration of Cu. Based on the BEM, size, shape, and relaxation, dependent CE of BNPs can be written as

(2) $$E_{c}=E_{c,b}\left({1-4\delta \alpha n}^{-1/3}\right){,\;n}^{-1/3}=d/D$$

where $E_{c,b}$ denotes the CE of bulk, *α* and *δ* are the shape and the relaxation factor, respectively. *n* is the total atom number of a nanoparticle; *d* and *D* represent the diameters of single atoms and particles. This manuscript only considers the effect of size on formation enthalpy. We estimated that the particle (the original particle of W and Cu) shape is spherical and each atom of the particle has 1/4 bond that is dangling, i.e., *α* = 1, *δ* = 1/4. Therefore, Equation (2) can be rewritten as

(3) $$E_{c}{=E}_{c,b}\left({1-n}^{-1/3}\right){,\;n}^{-1/3}=d/D$$

Replacing the Equation (3) into Equation (1), the formation enthalpy of the W–Cu nanoparticles can be written as

(4) $$H^{WCu}{=E}_{c,b}^{WCu}\left({1-n}^{-1/3}\right)-\left(1-x\right)E_{c,b}^{W}\left\{1-{\left[\left(1-x\right)n\right]}^{-1/3}\right\}{-xE}_{c,b}^{Cu}\left[1-{\left(xn\right)}^{-1/3}\right]$$

By Equation (4), the formation enthalpy of W–Cu nanoparticles can be calculated. The CE of bulk W–Cu alloys can be calculated by the modified analytic embedded-atom method (MAEAM) [20]. In this method, the CE of bulk W–Cu alloy can be written as

(5) $$E_{c,b}^{WCu}=\left[\frac{1}{2}{ø}^{W}\left(r\right){+F}^{W}\left(\rho \right){+M}^{W}\left(P\right)\right]\left(1-x\right)+\left[\frac{1}{2}{ø}^{Cu}\left(r\right){+F}^{Cu}\left(\rho \right){+M}^{Cu}\left(P\right)\right]x$$

where *ø* (*r*), *F* (*ρ*), and *M* (*P*) are the pair potential function, the embedding energy, and the modified term, respectively. *ρ* and *P* are second-order items of electron density. The detailed description of each item can be found in References [21,22]. Therefore, combining Equations (4) and (5), the formation enthalpy of W–Cu BNPs can be calculated. Meanwhile, when the formation enthalpy is equal to zero, we can calculate the value of the critical size (*D_c_*).

### 2.2. Experimental Procedures

**Figure 1:**
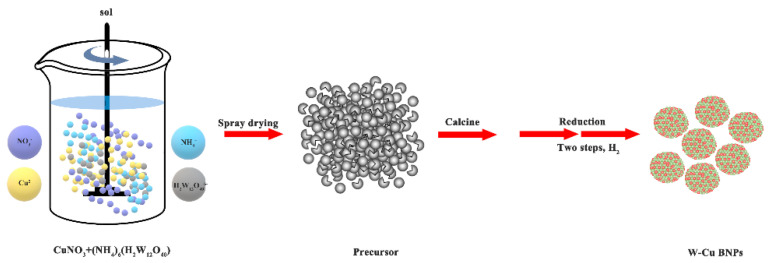
Synthesis schematic of W–Cu BNPs via a nano in situ composite method.

To verify our calculation results, we used the nano in situ composite method to prepare alloyed W50Cu50 BNPs. The preparation process is shown in Figure 1. Firstly, (NH_4_)_6_(H_2_W_12_O_40_)∙nH_2_O (AMT) and Cu(NO_3_)_2_∙3H_2_O were dissolved into deionized water according to the desired components (W50Cu50) and were stirred evenly at the same time. Then, the mixed solution was dried in a spray dryer at 200 °C for 1 h. Subsequently, these powders were calcined at 300 °C for 2 h to obtain the oxide mixture. Finally, the alloyed W50Cu50 BNPs were obtained by a two-step reduction process in a flowing hydrogen atmosphere; the first step was at 350–400 °C for 2 h and the second was at 750 °C for 2.5 h. It is worth noting that the nano in situ composite method is based on the concept of atomic-level mixing, so the initial synthesis size is in atomic scale.

TEM, HRTEM, HAADF, and EDS images of W–Cu BNPs were observed by aberration-corrected scanning transmission electron microscopy (STEM, FEI Titan G2 60–300, Hillsboro, OH, USA). All TEM were operated at an accelerating voltage of 200 kV.

## 3. Results and Discussion

**Figure 2:**
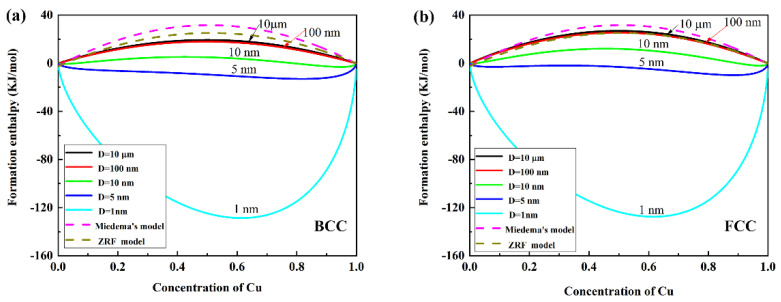
Formation enthalpy of W–Cu system as the function of Cu concentration at different particle sizes. (**a**) BCC crystalline structure, (**b**) FCC crystalline structure. $E_{b}^{W}$ = −336 kJ mol^−1^, $E_{b}^{Cu}$= −824 kJ mol^−1^, *d_W_*= 0.278 nm, *d_Cu_*= 0.2556 nm; all the data are taken from Reference [24].

Since the W–Cu system has a combination of different crystalline structures, we assume the W–Cu BNPs have two crystalline structures, i.e., face-centered-cubic (FCC) and body-centered-cubic (BCC). Figure 2 shows the relationship between synthesis size and formation enthalpy of W–Cu BNPs with different compositions and crystalline structures. To verify the accuracy of the BEM model used in this work, we also added curves calculated by the two other models, (Miedema’s model [8] and ZRF model [23]), represented by pink and brown dotted lines, respectively. In the absence of considering the influence of size on formation enthalpy, Miedema’s model and ZRF model can only calculate the formation enthalpy of bulk materials; that is, just the case of large-size synthesis can be calculated. In Figure 2, the results calculated by using the BEM model and the results calculated by Miedema’s model and ZRF model are basically consistent in the case of large-size synthesis. Naturally, the results of our calculation are credible.

**Figure 3:**
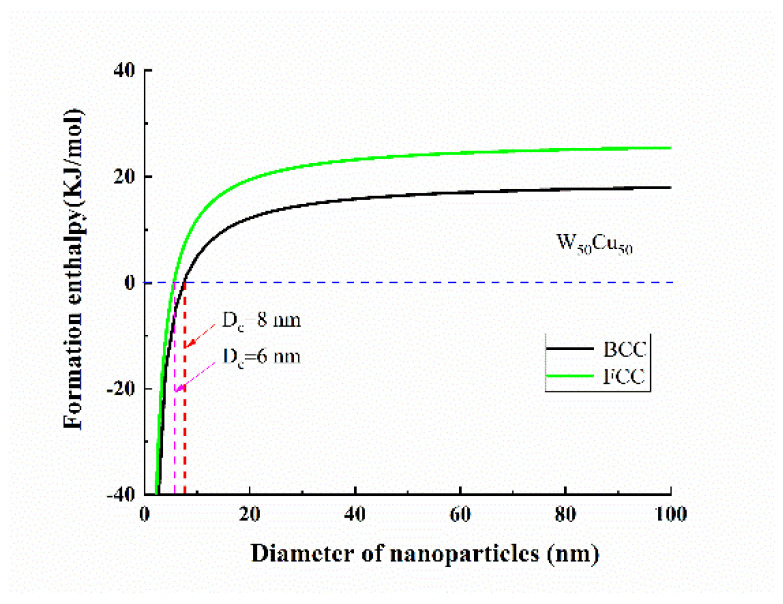
Formation enthalpy of W50Cu50 nanoparticles with different crystalline structure as the function of particle size, where *d_WCu_* = (*d_W_* + *d_Cu_*)/2 = 0.2668 nm.

As shown in Figure 2, the formation enthalpy of W–Cu system decreases rapidly as the particle size decreases. When the particle size is 10 μm, the value of formation enthalpy closes to bulk materials. When the particle size is less than 10 nm, the formation enthalpy changes from positive to negative. For example, as the particle size is 5 nm, the formation enthalpy within the full concentration region of Cu (0 < *x* < 1) is negative, which shows that the alloying of W and Cu nanoparticles becomes easy. From the thermodynamic views, the alloyed W–Cu BNPs within this size range have better stability. Thus, there should be a critical size (*D_c_*) between 5 and 10 nm to determine the alloying for W–Cu system. For W50Cu50 nanoparticles with different crystalline structures, the formation enthalpy as the function of different particle sizes is shown in Figure 3. When the particle size of W50Cu50 nanoparticles with BCC crystalline structure is below 8 nm, the formation enthalpy of this system turns from positive to negative and drops down rapidly. Thus, the D_c_ is 8 nm and 6 nm for BCC and FCC crystalline structures, respectively. Naturally, if the synthesis size is kept below the critical size, the stable and alloyed W–Cu BNPs can be obtained, even though they are immiscible in a bulk state.

**Figure 4:**
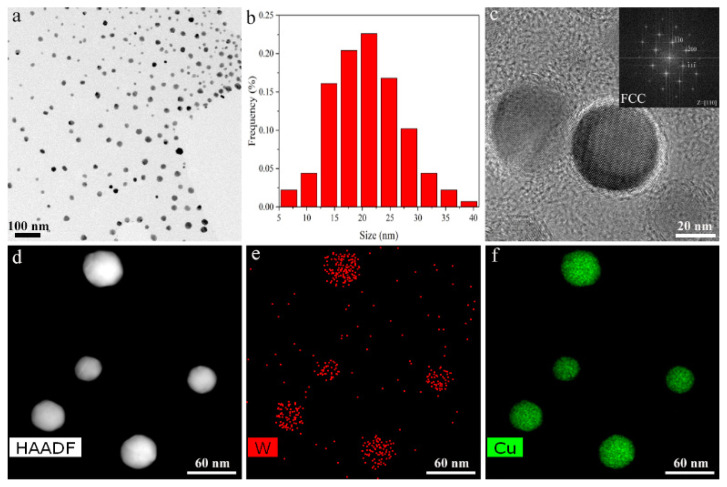
Characterization of microstructure and chemical composition of alloyed W50Cu50 BNPs. (**a**) TEM image of W50Cu50 BNPs, (**b**) size-distribution diagram of alloyed BNPs. (**c**) HRTEM image of W50Cu50 single-particle; inset is corresponding FFT pattern. (**d**) HAADF image of W50Cu50 BNPs. (**e**,**f**) EDS image of W and Cu element, respectively.

Based on the above prediction results, the synthesis size is reduced to below the critical value and alloyed W–Cu BNPs can be synthesized. Meanwhile, with the aid of the nano in situ composite method, we successfully synthesized alloyed W50Cu50 BNPs. Figure 4a shows the TEM image of W50Cu50 BNPs. The shape is nearly spherical, and the average particle size is less than 50 nm (Figure 4b). Figure 4c is the high-resolution transmission electron microscope (HRTEM) image of W50Cu50 BNPs. Inset is the corresponding fast Fourier transform (FFT) pattern of Figure 4c taken from along the [110] zone axis, which shows that the crystalline structure of W50Cu50 BNPs is face-centered cubic (FCC). In order to more intuitively identify the chemical composition, the STEM-EDS technology was adopted. Figure 4d is the partially enlarged high-angle annular dark-field (HAADF) image of Figure 4a. Figure 4e,f are the corresponding element maps of Figure 4d; they show that W and Cu are relatively uniformly mixed into a single structure of alloyed BNPs instead of a phase-separated structure. Therefore, the experimental results adequately verify our theoretical prediction that reducing the size to 10 nm can obtain alloyed W–Cu BNPs.

## 4. Discussion

The key to achieve alloying between immiscible elements lies in the competition between surface energy and formation enthalpy [25,26]. In the case of bulk synthesis, because the formation enthalpy is much greater than the surface energy, it is often only possible to form a mechanical mixture; there is also a case where the interface alloying may also occur with the help of dynamics under extreme conditions, and this phenomenon has been observed many times in experiments [7,27,28]. When the synthesis size is very small, especially when it is reduced to less than 10 nm, the surface energy is greatly increased, and the formation enthalpy is also reduced to a negative value. Driven by this dual effect, the alloying ability between immiscible elements is greatly improved. Specifically, due to the high surface-to-volume ratio and surface free energy, nanoparticles have a strong tendency to merge even at temperatures much lower than their melting points [29,30]. This is because gathering together can reduce the surface free energy, which is driven by the spontaneity of thermodynamics. This feature enables the W–Cu system with a large miscibility gap to be spontaneously alloying during the synthesis process with a nanoscale size. On the other hand, the synthesis of W–Cu BNPs by the nano in situ composite method is based on an atomic-scale mixing, and the original synthesis size is much smaller than 10 nm, so a single-phase solid solution W–Cu BNP can be obtained.

## 5. Conclusions

Based on the BEM model, we calculated the size effect on alloying ability of the immiscible W–Cu system. The results reveal that reducing the synthesis size can greatly enhance the alloying ability of the W–Cu system. For W50Cu50 BNPs, the critical sizes of alloying are 6 and 8 nm for FCC and BCC crystalline structures, respectively. At the same time, the atomic-scale alloyed W50Cu50 BNPs with an average size of less than 50 nm were successfully prepared according to the nano in situ composite method. The experiment results have well confirmed the theoretical prediction. As a consequence, we believe the nano in situ composite method is useful for the preparation of alloyed BNPs between immiscible elements.
